# Proline Metabolism in Malignant Gliomas: A Systematic Literature Review

**DOI:** 10.3390/cancers14082030

**Published:** 2022-04-17

**Authors:** Magdalena M. Sawicka, Karol Sawicki, Tomasz Łysoń, Barbara Polityńska, Wojciech Miltyk

**Affiliations:** 1 Department of Analysis and Bioanalysis of Medicines, Medical University of Bialystok, Mickiewicza 2D, 15-222 Bialystok, Poland; wojciech.miltyk@umb.edu.pl; 2 Department of Neurosurgery, Medical University of Bialystok, Skłodowskiej-Curie 24A, 15-276 Bialystok, Poland; karol.sawicki@umb.edu.pl (K.S.); tomasz.lyson@umb.edu.pl (T.Ł.); 3 Department of Psychology and Philosophy, Medical University of Bialystok, Szpitalna 37, 15-295 Bialystok, Poland; bpolitynska@wp.pl

**Keywords:** glioblastoma, glioma, astrocytoma, oligodendroglioma, proline, prolidase, proline oxidase, proline dehydrogenase, pyrroline-5-carboxylase

## Abstract

**Simple Summary:**

Studies of various types of cancers have found proline metabolism to be a key player in tumor development, involved in basic metabolic pathways, regulating cell proliferation, survival, and signaling. Here, we systematically searched the literature to find data on proline metabolism in malignant glial tumors. Despite limited availability, existing studies have found several ways in which proline metabolism may affect the development of gliomas, involving the maintenance of redox balance, providing essential glutamate, and affecting major signaling pathways. Metabolomic profiling has revealed the importance of proline as a link to basic cell metabolic cycles and shown it to be correlated with overall survival. Emerging knowledge on the role of proline in general oncology encourages further studies on malignant gliomas.

**Abstract:**

Background: Proline has attracted growing interest because of its diverse influence on tumor metabolism and the discovery of the regulatory mechanisms that appear to be involved. In contrast to general oncology, data on proline metabolism in central nervous system malignancies are limited. Materials and Methods: We performed a systematic literature review of the MEDLINE and EMBASE databases according to PRISMA guidelines, searching for articles concerning proline metabolism in malignant glial tumors. From 815 search results, we identified 14 studies pertaining to this topic. Results: The role of the proline cycle in maintaining redox balance in IDH-mutated gliomas has been convincingly demonstrated. Proline is involved in restoring levels of glutamate, the main glial excitatory neurotransmitter. Proline oxidase influences two major signaling pathways: p53 and NF- κB. In metabolomics studies, the metabolism of proline and its link to the urea cycle was found to be a prognostic factor for survival and a marker of malignancy. Data on the prolidase concentration in the serum of glioblastoma patients are contradictory. Conclusions: Despite a paucity of studies in the literature, the available data are interesting enough to encourage further research, especially in terms of extrapolating what we have learned of proline functions from other neoplasms to malignant gliomas.

## 1. Introduction

In the adult population, glioblastoma (GBM), astrocytoma grade 3 and 4, and oligodendroglioma grade 3 are the most common primary malignant brain tumors [1,2]. While contributing to only 2% of all malignancies, gliomas cause a disproportionate amount of cancer-related morbidity and mortality. Even with optimal treatment, the median survival following a diagnosis of GBM is no more than 2 years [3,4]. Despite tremendous efforts, for almost two decades after introducing the current treatment regimens, there has been no significant breakthrough in therapy [5].

The role of proline has been shown to be prominent in the biology of several neoplasms because of the significant influence that proline exerts on life-defining metabolic cycles, i.e., the tricarboxylic acid cycle (TCA), glycolysis, the urea cycle, and the pentose phosphate pathway (PPP) [6,7]. Additionally, in recent years, ample evidence has been found linking proline metabolism with crucial regulatory pathways, e.g., amino acid response (AAR), phosphatidylinositol 3-kinase (PI3K)/protein kinase B (Akt), mammalian target of rapamycin (mTOR), extracellular signal-regulated kinase (ERK), the Janus kinase/signal transducers and activators of transcription (JAK/STAT), and ROS-driven signaling [8]. In contrast to general oncology, our knowledge pertaining to the role of proline in the biology of malignant gliomas is limited. Nevertheless, it would appear that the time is ripe to consider proline metabolism in gliomas. Specifically, it has been already shown that mutation of IDH—an enzyme participating in the TCA cycle—results in the accumulation of D-2 hydroxyglutarate (2-HG), which is a typical oncometabolite, but is produced from αKG, which in turn is a metabolite of proline [9,10,11,12]. Thus, proline-focused insight into glioma biology might provide a promising extension to our knowledge, with the potential to discover new and hopefully more effective therapies for these hitherto unconquerable tumors.

### Proline—“An Essential Non-Essential Amino Acid”

**Figure 1:**
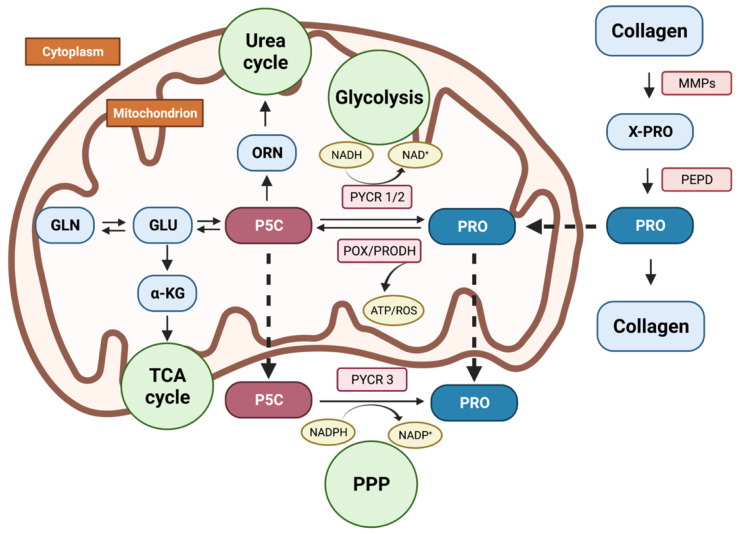
Proline cycle and its basic metabolic links in the human cell. Proline released during collagen catabolism can feed back to collagen resynthesis, or, through the proline cycle, it may be used for energetic purposes, redox balance, parametabolic regulation, or anaplerosis. PRO—proline, P5C—pyrroline-5-carboxylate, ORN—ornithine, GLN—glutamine, GLU—glutamate, αKG—alpha-ketoglutarate, PEPD—prolidase, POX/PRODH—proline oxidase/proline dehydrogenase, PYCR 1/2/3—pyrroline-5-carboxylate reductase 1/2/3, MMPs—metalloproteinases, PPP—pentose phosphate pathway, TCA cycle—tricarboxylic acid cycle. Created with BioRender.com, accessed on 2 February 2022.

The metabolism of proline (Figure 1) is uncoupled from other non-essential amino acids (NEAAs) because of the unique, cyclic structure of the alpha-amino group contained within a pyrrolidine ring. Therefore, proline cannot be processed with standard decarboxylases, aminotransferases, and racemases but depends on a dedicated set of enzymes [13,14]. Given their strategic position, the enzymes of proline metabolism become both targets and effectors in several regulatory mechanisms [15].

The only enzyme that is capable of cleaving proline from the dipeptide C-terminus is prolidase (PEPD). Prolidase is expressed ubiquitously and has an essential role in the turnover of collagen, up to 25% being built from proline [16]. Genetic defects affecting prolidase, such as prolidase deficiency in autosomal recessive mutations of the *PEPD* gene, affect organs that are highly dependent on the quality of collagen, i.e., the skin and musculoskeletal system. Prolidase deficiency also manifests with severe mental retardation, hypothetically due to impaired proline turnover resulting in failed neural migration during the development of the central nervous system (CNS) [17,18]. High levels of prolidase activity in the hippocampus and cerebellum have been reported in adult male rat brains. Elevated prolidase activity has been found to induce N-methyl-D-aspartate (NMDA) receptor dysregulation by increasing plasma proline levels in schizophrenia. On the other hand, decreased prolidase activity in patients with post-traumatic stress disorder has been associated with hippocampal damage [19].

**Figure 2:**
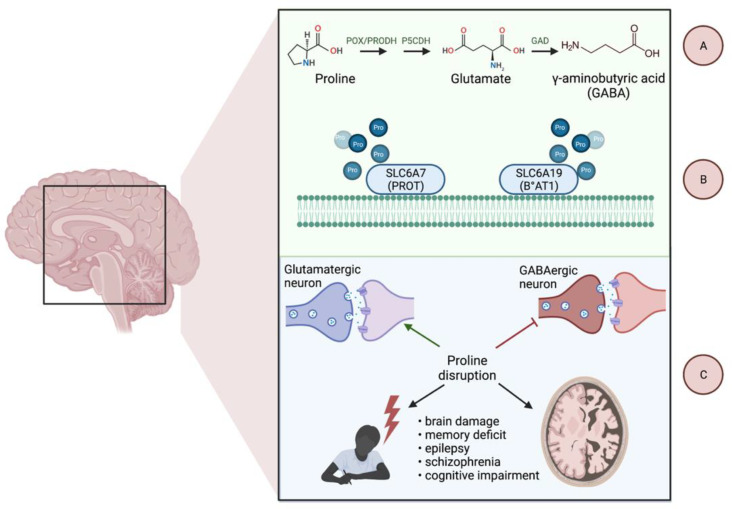
The role of proline in brain function. (**A**) Proline is a forerunner of key CNS neurotransmitters, glutamate, and GABA. (**B**) Therefore, proline concentration in the synaptic cleft is precisely controlled by dedicated transporters. (**C**) Disturbances in proline metabolism affect both the developing and the adult brain, causing neurotoxic effects. GAD—glutamate decarboxylase. Created with BioRender.com, accessed on 2 February 2022.

Further catabolism of proline takes place in the mitochondrial matrix by means of the flavin-dependent enzyme proline oxidase/proline dehydrogenase (POX/PRODH). The enzyme, through flavin adenine dinucleotide (FAD), donates electrons through ubiquinone to the electron transport chain (ETC), producing ATP, or electrons are transferred directly to the oxygen, thus formulating reactive oxygen species (ROS). The outcome of this process—pyrroline-5-carboxylate (P5C)—remains in spontaneous equilibrium with glutamate-γ-semialdehyde (GSAL). In reverse, P5C is the only possible precursor to proline that may be reduced solely by the nicotinamide adenine dinucleotide (phosphate) (NAD(P)^+^)-dependent enzymes from the pyrroline-5-carboxylate reductase family (PYCRs), that is, PYCR1 and PYCR2 located in the mitochondria and PYCR3 (aka PYCRL) found in cytoplasm [20,21]. Proline, as a substrate easily accessed from catabolized collagen, might be incorporated into several major metabolic pathways. α-Ketoglutarate (αKG), derived from proline, replenishes the TCA cycle in a process called anaplerosis [22]. The proline–P5C interchange between the cytosol and the mitochondria, called the proline cycle, works as a shunt that allows for NAD(P)H inflow to the oxidative chain and provides the PPP with the reducing potential required in nucleotide synthesis [7,23]. P5C, as a tautomer of GSAL, is a direct forerunner of glutamine and gamma-aminobutyric acid (GABA) and might be reversibly converted to ornithine that enters the urea cycle to produce arginine, citrulline, creatine, or polyamines [24,25]. Of note, both GABA and glutamate are essential CNS neurotransmitters, and the latter is considered the main excitatory glial stimulant, both in the healthy brain and in glial neoplasms [26]. Additionally, proline itself displays some neurotransmitter-like features e.g., an ability to activate the NMDA or α-amino-3-hydroxy-5-methyl-4-isoxazilepropionic acid receptor (AMPA). Proline concentration is selectively regulated by proline transporters—solute carrier family 6 member 19 (SLC6A19, B◦AT1) and solute carrier family 6 member 7 (SLC6A7, PROT) [27,28], which belongs to the family of dopamine, serotonin, norepinephrine, and glycine transporters and shares with them 40–50% of sequence identity [29]. An elevated concentration of proline is known to exert neurotoxic effects and impair the glutamate- and GABA-dependent functions of synapses [30]. In addition, there are well-documented associations between proline and schizophrenia [31], Huntington’s, Parkinson’s, and Alzheimer’s diseases, as well as for inherited hyperprolinemia presenting as psychiatric disorders, brain damage, and spatial memory deficits [28,30,32,33] (Figure 2).

The metabolism of proline and the biology of malignant glial tumors might be linked at multiple levels. One objective of this review is to summarize the current state of knowledge available on the topic and to highlight some areas in which further research might be particularly interesting for understanding the pathogenesis of gliomas.

## 2. Materials and Methods

Following PRISMA guidelines, a systematic review of the literature was performed to identify studies focused on proline metabolism in malignant gliomas. The MEDLINE and EMBASE databases (https://pubmed.ncbi.nlm.nih.gov, Scopus accession date: 31 January 2021) were searched using combinations of terms concerning tumors—“glioblastoma”, “glioma”, and “high grade glioma”—and proline metabolism (combined with the Boolean “OR” operator)—“proline”, “proline oxidase”, “POX”, “proline dehydrogenase”, “PRODH”, “pyrroline-5-carboxylate reductase”, “PYCR”, “P5C reductase”, “prolidase”, “peptidase D”, and “imidodipeptidase”. Since a systematic literature review is not eligible for PROSPERO registry, the study has been registered on Open Science Framework (DOI: 10.17605/OSF.IO/43N7X).

## 3. Results

**Figure 3:**
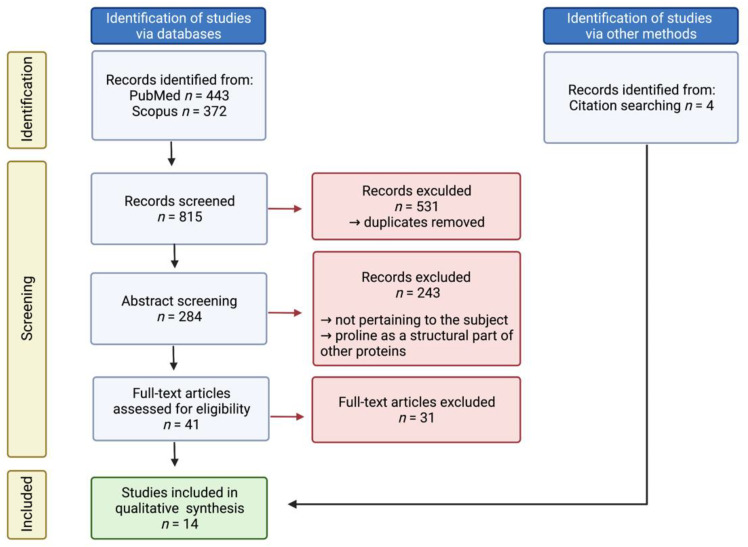
Flowchart of the systematic literature review and meta-analysis according to the PRISMA guidelines. Created with BioRender.com, accessed on 2 February 2022.

A total of 815 full-text studies published in the English language before January 2021 were screened to determine eligibility for inclusion. Additional studies were found by reviewing the references of the included publications. Specifically, all studies regarding proline only as a structural part of other proteins, e.g., the active site of an enzyme, were excluded. Out of 45 articles chosen for a thorough analysis, 14 were eventually identified as pertaining to the scope of the study. A flow chart (Figure 3) and a list of all included studies with a short summary (Table 1) are presented.

## 4. Discussion

### 4.1. Historical Studies on Gliomas

In a study published in 1976, Lefauconnier et al. [47] compared 13 samples of glioblastoma with 13 normal brain biopsy specimens, concluding that the concentration of proline in GBM was tenfold higher than that in the normal brain, and this was the highest difference among all of the substances evaluated. This was the first study to signal the relevance of proline metabolism in GBM. By contrast, in another historical study by Loreck et al. published in 1987 [46], the authors conducted a series of experiments on human astrocytes and glial tumor cell lines. While manipulating extracellular concentrations of proline and P5C, the authors found no changes in cellular metabolism. Additionally, there was no difference in PYCR activity between glia and neoplasm cell cultures, and the activity of POX/PRODH was not detected. Thus, the authors concluded that the proline cycle does not function either in glial cells or in GBM. We hypothesize that the results of the latter study were decisive in discontinuing research on proline metabolism in glial tumors for the next 30 years.

### 4.2. Role of Proline Metabolism in Gliomas

However, a recent, methodologically excellent study by Hollinshead et al. [34] came to somewhat different conclusions. The researchers demonstrated that in anaplastic oligodendroglioma (grade 3) bearing the IDH mutation, the proline cycle plays a significant role. By means of the proline shuttle mechanism described by Hagedorn and Phang in the 1980s [48,49], the IDH-mutant gliomas can maintain redox balance, uncouple the TCA cycle from respiration, and provide an efficient detour for oxidative ATP production while sparing oxygen. As suggested in the article, this particular feature of IDH-mutated oligodendroglioma is a stress response that allows for survival in a hypoxic environment, which is regarded as a characteristic feature of gliomas, particularly GBM. In the conducted experiment, glutamate was the main source of proline, incorporated via GSAL and P5C. This is another important point linking proline metabolism with glutamate, the main CNS excitatory neurotransmitter, which plays a crucial role in GBM and, for that reason, is dubbed the “glutamate-addicted” tumor.

The work of Cappelletti et al. [35] provided additional evidence on the links between proline and glutamate metabolism in GBM. The researchers used the U87 GBM cell line with wild-type and lessened-activity POX/PRODH in order to track changes in the intracellular concentration of glutamate and the effects on cell viability. Indeed, there was a significant drop in the glutamate concentration in POX/PRODH-impaired cells; however, the cells were able to compensate for the deficit in glutamate within 72 h, and that ability might explain why there were no changes in their viability. However, it is worth keeping in mind that direct extrapolations of results from cell culture studies to clinical practice might be biased. The metabolic profiles of surgically resected and in vitro cultured gliomas have been shown to be significantly different, with the influence of the tumor microenvironment perceived as a key factor.

In a study by Panosyan et al. [44], the researchers confirmed the contribution of POX/PRODH to the clinical course of GBM. Using maps of gene expression from large datasets (540 samples), they found that increased POX/PRODH expression, which was present in exactly half of the cases, was associated with shortened overall survival. Additionally, POX/PRODH expression was found to be lower in GBM patients than in controls. Although significant, these findings were discussed without greater elaboration as to their significance.

A connection between proline and arginine metabolites/metabolic pathways was shown to be significant in studies on the metabolomic profiling of glioma tissue, plasma samples, or microdialysis fluid. In a study by Björkblom et al. [42], the authors examined microdialysis fluid derived from recurrent glioblastomas before and during interstitial treatment with cisplatin and from the adjacent non-affected brain. Metabolomic profiling of microdialysis fluid revealed that the concentration of proline was significantly different in glioblastoma as opposed to the adjacent brain tissue.

A study by Zhao et al. [40] found that proline and arginine metabolites were reliable discriminators between gliomas: low- vs. high-grade and IDH-mutant vs. wild-type. Interestingly, whereas proline plasma concentration was not altered by IDH mutation status, the metabolites closely linked to P5C, e.g., ornithine and arginine, were found to differ significantly between high- and low-grade gliomas, though no precise discrimination was made as to which of these metabolites was the most important marker. It is noteworthy that among all the pathways analyzed, the authors found that those involved in arginine and proline metabolism were of the greatest significance in differentiating between malignant and so-called low-grade gliomas, as in the case of IDH mutation status. Huang et al. [41] conducted an analysis of plasma metabolites in GBM patients in relation to matched controls. As in the work of Zhao et al. [40], the concentration of metabolites involved in the proline and urea cycle, i.e., 2-oxoarginine, argininate, and N-acetylarginine, were found to be significantly different between the two groups. The authors also concluded that plasma proline concentration has the potential to serve as a glioma biomarker prior to diagnosis. The work of Jonsson et al. [43] implements machine learning algorithms into the analysis of plasma metabolomic profiles in a large database containing multiple samples collected prospectively. The authors found that among other amino acids, proline concentration was significantly altered in glioblastoma patients. Additionally, by analyzing samples obtained prior to glioma diagnosis, plasma proline concentration was shown to be useful as a pre-diagnostic marker. A study by Prabhu et al. [45] integrated data from glioma metabolomic and genetic profiles. Analysis of a database consisting of fresh-frozen tumor samples led the authors to the conclusion that the metabolic pattern of several amino acids, including proline, was significantly altered. The authors associated the observed variability with aberrant tumor metabolic reprogramming leading to tumor heterotrophy, i.e., an adaptation to exploit alternative sources of nutrients.

Although the aforementioned metabolomic studies found proline and its metabolism to be relevant in the pathogenesis of gliomas, they did not discuss possible reasons for the dysfunction nor any implications that may arise from them.

Overall, the evidence supports the anaplerotic and parametabolic role of proline in glioma metabolism. The proline cycle is crucial in tumors with the IDH-mutation, which is a well-known favorable prognostic factor, making it a promising therapeutic target. The involvement of proline in the urea cycle and glutamate metabolism has been shown to be relevant but warrants further investigation.

### 4.3. Regulatory Role of Proline Metabolism in Gliomas

Shao et al. [39] conducted a series of experiments elucidating the molecular mechanism of p53 regulation of POX/PRODH in GBM cell cultures and in an animal model. In this context, POX/PRODH, which is in the p53-dependent transcriptome, was deemed to have tumor-suppressing activity. In a study on C6 GBM cell lines incubated with different concentrations of proline, Ferreira et al. [36] found a significant increase in both ROS and nuclear factor kappa B (NF-κB) activity correlated with increasing quantities of proline and thus hypothesized that proline may favor signaling toward cell proliferation. This result might be explained in terms of potentially increased POX/PRODH activity, known for generating ROS; however, this was not measured by the authors.

The studies of Gönullu et al. and Verma et al. provided contradictory results on the effects of prolidase activity in GBM patients vs. controls [37,38]. Whereas the experiment conducted by the Gönullu group found that plasma prolidase activity was lower in GBM, Verma et al. found the opposite. Additionally, the latter study provided a measurement of prolidase activity in tissues, i.e., excised GBM vs. healthy brain, indicating that prolidase activity is consistently higher in GBM than that measured in the plasma.

### 4.4. Missing Links between Proline Metabolism and Gliomas

**Figure 4:**
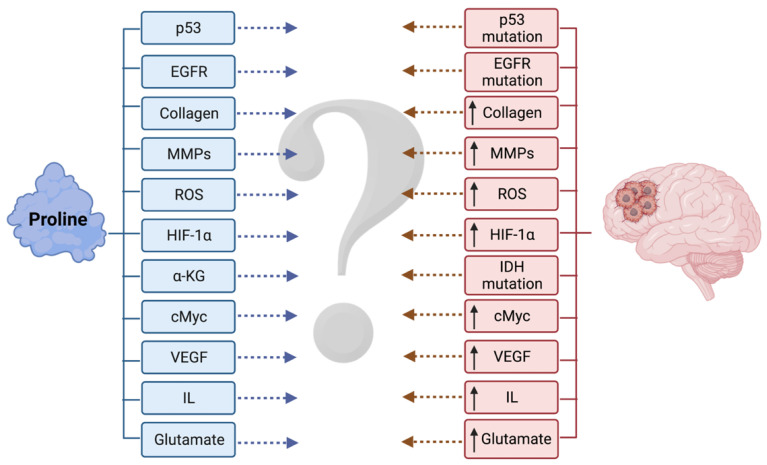
Malignant gliomas are still one of the most enigmatic clinical entities. Although our knowledge of proline metabolism in tumors is constantly expanding, synthesis of data pertaining to both of these domains is lacking. ↑ upregulated. Created with BioRender.com, accessed on 2 February 2022.

Because the data on gliomas are limited, there is a need to extrapolate the regulatory mechanisms of proline metabolism known from non-glial tumors to those of gliomas in order to determine if they continue to hold true (Figure 4).

Prolidase has been recognized as an essential factor shaping the extracellular matrix (ECM), especially in collagen. ECM is not only a scaffold providing adhesion sites for cells but is also a major force driving cell migration, regeneration, and maintenance of synaptic connections [50,51]. Prolidase deficiency, both in humans and in knocked-out mice, is associated with the symptoms of mental retardation as a consequence of impaired neuron migration during brain development, which is executed by means of the extracellular matrix and adhesion receptors, all affected by collagen turnover problems [52]. In contrast to a healthy brain, which contains relatively small amounts of collagen (mostly type IV collagen involved in the basement membranes of pia meninx or vascular endothelial cells), gliomas are known for the synthesis of type IV and XVI collagen into the extracellular matrix [53]. Moreover, diffuse gliomas are known for primary infiltrative growth into the brain, with tumor cells migrating along ependyma, white matter fibers, and vessels [54]. The evidence emphasizes the role of the extracellular matrix in tumor invasiveness and prognosis. On the other hand, the excessively rapid metabolic rate, particularly in the case of GBM, cannot be followed by appropriate neovascularization and eventually results in necrosis [55,56], with collagen possibly serving as a reservoir of nutrients. Therefore, one might expect that in an area of rapid tumor growth, where catabolic activity might be increased in order to take advantage of the nutrients pooled in the collagen, prolidase activity might also be elevated.

In the regulatory domain, prolidase has the well-recognized ability to activate epidermal growth factor receptor (EGFR), resulting in the triggering of dependent downstream kinase systems, such as PI3K/Akt, mTOR, ERK, and JAK/STAT3, and, overall, in the transcription of genes associated with the growth, differentiation, and proliferation of cells [16]. Amplifications and mutations in EGFR have been detected in approximately 45–60% of GBM cases studied and have been related to GBM pathogenesis and resistance to treatment [57,58]. Similarly, prolidase has an affinity for human epidermal growth factor receptor 2 (HER2, ErbB2), producing activation of downstream tyrosine kinases, presenting oncogenic activity [16]. The HER-2 mutation is associated particularly with a secondary GBM, detected in up to 40% of cases [59]. Prolidase has also been found to be a key regulator of p53 activity. More than half of cytoplasmic and nuclear p53 is inactivated by temporary binding with prolidase. Under conditions of cellular stress or prolidase deficiency, the prolidase-p53 complex dissociates, which activates p53 and its pro-apoptotic potential [60]. Using data from the Cancer Genome Atlas Research Network, it has been estimated that 87% of GBM cases had alterations in the p53 signaling pathway, with 28–35% of cases having deleted or mutated p53 [59].

Further, high cytoplasmic concentrations of proline itself are known to exert a regulatory influence on hypoxia-inducible factor-1α (HIF-1α) transcriptional activity. Up-regulated HIF-1α activates dependent pathways, such as COX-2, vascular endothelial growth factor (VEGF), tumor necrosis factor α (TNF-α), transforming growth factor β (TGF-β), interleukin-1 (IL-1), NF-κB [61], and glucose transporter-1 (GLUT-1) expression, and therefore induces angiogenesis, controls proliferation and differentiation, and influences the uptake of glucose [62]. Tumor hypoxia is a hallmark of malignant gliomas, and it has been demonstrated in vivo and in vitro that glioma cells overexpress HIF-1α, which results in the activation of VEGF or MMPs. Additionally, there exists some evidence that this overexpression may occur even in the absence of hypoxia because of genetic alterations encountered in malignant gliomas [63,64]. Moreover, glioma cells exert a paracrine influence on the surrounding environment, leading to the “re-education” of glioma-associated microglia. This renounces the antitumor activity and contributes to glioma invasion, releasing multiple cytokines, i.e., IL-1β, IL-6, IL-8, IL-10, and TNF-α [65,66]. Proline has been shown to activate the TGF-β1 receptor. There is also an interesting mutual relationship between prolidase; its activity results in proline concentration and TGF-β1-dependent mTOR kinase functioning [16]. TGF-α and TGF-β secreted in greater amounts by glioma cells are known to favor the communication between these cells and activated astrocytes found within and around the tumor [67,68].

Given the aforementioned results of the studies by Cappelletti et al. and Panosyan et al. [35,44], POX/PRODH seems to play a contradictory role in GBM. The data from other non-glial neoplasms corroborates the inconclusive role of POX/PRODH in the studies we examined [8,69]. On the one hand, POX/PRODH might act as a tumor survival factor through AMPK kinase or peroxisome proliferator-activated receptors-γ (PPARγ) when induced under stress conditions, e.g., in nutrient or oxygen deprivation or stimulation by oxidized lipids (oxLDL) [13,70,71]. On the other hand, POX/PRODH is believed to be a potent tumor-suppressor when activated by p53 or PPARγ in conditions related to DNA damage or during inflammation processes. Additionally, ROS that are generated as a consequence of POX/PRODH enzymatic activity might act in two opposite directions as well, directing the cell either towards apoptosis or prosurvival autophagy; however, the “switch” that changes the cell route remains unknown [62,72]. It has been demonstrated that the overexpression of EGFR variant III (EGFRvIII) leads to elevated ROS levels in GBM and facilitates further alterations in the genome of GBM cells [73]. Of note, POX/PRODH is inhibited by both the c-Myc oncogene and its product, miR23b* [74]. C-Myc levels are strictly correlated with the malignancy grade of gliomas, and about 60–80% of GBM display elevated c-Myc levels [75]. Furthermore, c-Myc encourages the Warburg effect, drives glycolytic flux, and elevates intratumoral levels of glutamine in GBMs [76].

### 4.5. Ambiguous Role of POX/PRODH in General Oncology

A number of studies utilizing various types of neoplasms have provided ample evidence in support of proline being a key player in rewired tumor metabolism, displaying several regulatory, anaplerotic, and parametabolic properties. The first experiments conducted on colorectal cancer cell lines and in the mouse xenograft model led to the discovery that POX/PRODH is p53-induced gene-6 (PIG-6), which acts as a tumor suppressor, and this suppression depends on the quantity of the enzyme—high levels of POX/PRODH expression lead to apoptosis, while lower levels cause cell cycle arrest in the G2 phase and inhibit proliferation [8,77]. The POX/PRODH expression level was correlated with better prognoses in estrogen receptor-positive (ER^+^) breast cancer patients [78] and with apoptosis induction in MCF-7 cells [79]. Increased expression of POX/PRODH exerted a reciprocal inhibitory effect on the expression of the cyclooxygenase-2 (COX-2) enzyme [80], counteracting the prostaglandin-driven development of an inflammatory tumor milieu known for worsening the prognosis of several malignancies. In the model of oral squamous cell carcinoma, Tołoczko-Iwaniuk demonstrated that celecoxib, a well-known inhibitor of COX-2, increased levels of POX/PRODH and, as a result, led to apoptosis [81]. The anti-tumor properties of POX/PRODH, induced through stimulation by propolis, were also demonstrated in tongue squamous cell carcinoma cells (CAL-27) [82].

On the other hand, Olivares et al. showed that pancreatic ductal adenocarcinoma cells (PDAC) use proline derived from collagen as an alternative source of energy to fulfill the excessive metabolic requirements of the tumor. Additionally, the researchers demonstrated that the expression of POX/PRODH promotes tumor growth in vivo and in vitro [83]. Liu et al. [84] demonstrated the contribution of POX/PRODH in the progression of non-small cell lung cancer (NSCLC) through increased expression of pro-inflammatory cytokines that are associated with poor prognoses in NSCLC patients. Yan et al. [85] demonstrated that the expression of POX/PRODH is upregulated in prostate cancer (PCa), with a significant relationship between POX/PRODH expression and progression of the disease. In this case, POX/PRODH inhibits T-cell proliferation and function, as well as the production of ROS, eventually resulting in less restriction of tumor growth. The importance of POX/PRODH as a pro-tumor agent was also highlighted in breast cancer, particularly in breast cancer metastases. Elia et al. revealed the increased proline catabolism in metastases tissue compared to primary breast cancer patients [86]. In a study on triple-negative breast cancer (TNBC) treated with histone deacetylase (HDAC) inhibitors, Fang et al. [87] discovered that the induction of POX/PRODH played an anti-apoptotic role, while the lack of the enzyme significantly increased HDAC inhibitor-induced apoptosis. Some authors, attempting to summarize studies based on research in renal, rectal, stomach, and liver cancers [77,88], hypothesized that tumors need to down-regulate POX/PRODH activity in order to thrive [13].

Overall, POX/PRODH is considered a double-faced enzyme because depending on the specific circumstances, it may either promote tumor progression or lead to apoptosis and autophagy of cancer cells [62].

### 4.6. Pro-Neoplastic Role of PYCR in General Oncology

PYCR genes are identified as some of the most frequently upregulated in neoplasms in general, as found in a metabolomic analysis of a large dataset of more than 1900 various types of tumors [89]. For instance, a series of studies has confirmed the role of PYCR in lung cancer. NSCLC overexpresses PYCR1, and the expression is related to the patient’s clinical status and the progression of the disease. Wang et al. [90] confirmed that upregulated PYCR1 promoted the progression of NSCLC by activating the mitogen-activated protein kinase 13 (p38) pathway, while it was suppressed by microRNA-488 (miR-488). The work of Cai et al. [91] provided evidence that PYCR1 promotes the cell cycle and inhibits apoptosis by regulating cyclin D1, B-cell lymphoma-2 (Bcl-2), and B-cell lymphoma-extra large (Bcl-xl) expression in NSCLC. Additionally, Sang et al. [92] found that PYCR1 accelerated NSCLC metastasis spread by promoting epithelial–mesenchymal transition (EMT) pathways. Gao et al. [93] found multiple relevant connections in PYCR1 expression of lung adenocarcinoma (LUAD), e.g., in the metabolomic profile, the activity of signaling pathways, the progression of the disease, and overall patient survival times. PYCR1 was also significantly more highly expressed in breast cancer. Ding et al. [94] corroborated the finding that high PYCR1 expression correlates with a poor prognosis for breast cancer patients, independent of their estrogen receptor (ER) status. Moreover, the inhibition of PYCR1 reduced tumor growth and invasion capabilities, simultaneously sensitizing cancer cells to chemotherapy with doxorubicin. Zhuang et al. [95] documented the findings that PYCR1 is overexpressed in hepatocellular carcinoma (HCC) and that PYCR1 interference inhibits tumor growth and promotes apoptosis. Similar findings were reported by Ding et al. [96], who found that PYCR1 was one of the most upregulated enzymes in HCC and that its inhibition led to a reduced tumor burden. With regard to PCa, Zeng et al. [97] conducted an experiment in which the suppression of PYCR1 led to the inhibition of proliferation and colony formation, cell cycle arrest, and cell apoptosis, thus leading to the conclusion that the progression of PCa is dependent on the activity of PYCR1. In gastric cancer (GC), Xiao et al. [98] showed that the overexpression of PYCR1 enhanced tumor proliferation. Additionally, PYCR1 was upregulated because of glucose deprivation in a concentration- and time-dependent manner, and the PI3K/Akt pathway affected proline metabolism via PYCR1. Du et al. [99] demonstrated that PYCR1 is upregulated in bladder cancer, and the knockdown of the enzyme reduced tumor growth via the downregulation of Akt/Wnt/β-catenin signaling. Furthermore, Ye et al. [100] revealed that PYCR1 led to tumor growth via the Akt pathway in human malignant melanoma in vitro. In general, PYCR seems to act as a pro-neoplastic agent in a number of tumors, whilst the role of POX/PRODH is more ambiguous.

### 4.7. Prolidase Supports Tumor Metabolism—General Oncology

Aberrations in collagen metabolism and hence increased prolidase activity have been well-documented in certain cancers. It has been suggested that enhanced prolidase activity significantly influences the remodeling of the tumor microenvironment, and prolidase activity is correlated with the invasiveness of the cancer phenotype [101]. Plasma prolidase activity (PPA) was significantly higher in patients with gastric cancer not amenable to surgical resection than in operable cases and the control group. A strong correlation was found between tumor volume and PPA [102]. Prolidase activity was found to be significantly increased in breast cancer tissue, suggesting that the collagen turnover rate was increased in tumor tissue compared with the control [103]. Arioz et al. [104] demonstrated a significant increase in prolidase activity and oxidative stress in patients with endometrial cancer, which, according to the authors, may be related to local invasion of cancer. Gecit et al. [105] reported that increased levels of prolidase seemed to be associated with increased NO levels and oxidative stress, along with decreased antioxidant levels, in bladder cancer. According to a study by Guszczyn et al. [106], the expression of beta(1) integrin and prolidase activity were significantly elevated in stomach cancer, as was an enhancement in collagen turnover. Sayir et al. [107] conducted a study on esophageal cell carcinoma patients, seeking prognostic factors. The results revealed that prolidase activity, nitric oxide, total oxidant status, and oxidative stress index were increased, and the values were higher with more advanced stages of cancer. On the other hand, in pancreatic cancer, Palka et al. [108] observed the opposite tendency, in which prolidase activity was decreased.

This somewhat exclusive position among other NEAAs makes the enzymes of proline metabolism, i.e., prolidase, POX/PRODH, and PYCR, appropriate targets for regulatory functions. Indeed, a number of studies have indicated a multitude of connections between the aforementioned enzymes and major regulatory pathways, but knowledge about these relationships in gliomas remains limited.

## 5. Conclusions

Although the literature in the field is limited, there are a number of areas where the metabolism of proline and that of gliomas might be interlocked, both as a result of it being a versatile substrate on the crossroads of major pathways and its diverse regulatory properties. Further laboratory and clinical studies are warranted to understand the pathogenesis and support the exploration of potential therapies for gliomas.

## Figures and Tables

**Table 1:** A summary of 14 articles chosen for systematic review.

Author	Year	Title	Materials/Methods	Highlights
Hollinshead et al. [34]	2018	Oncogenic IDH1 Mutations Promote Enhanced Proline Synthesis through PYCR1 to Support the Maintenance of Mitochondrial Redox Homeostasis	Cell cultures: human anaplastic oligodendroglioma (grade 3) wild-type and IDH-mutant	IDH-mutant high-grade oligodendroglioma utilizes proline cycle as a redox shuttle to maintain redox balance. PYCR1 expression is increased in IDH1-mutated gliomas. Glutamine-derived proline synthesis is increased. An adaptation for hypoxic conditions: sparing oxygen allows for anabolism.
Cappelletti et al. [35]	2018	Proline oxidase controls proline, glutamate, and glutamine cellular concentrations in a U87 glioblastoma cell line	Cell cultures: human U87 GBM wild-type and POX-mutant	U87 human GBM cells with wild-type and impaired-activity POX/PRODH were studied to assess connections between proline and glutamate metabolism. A decrease in POX/PRODH activity caused a transient drop in proline, glutamate, and glutamine concentration.
Ferreira et al. [36]	2020	Effect of Proline on Cell Death, Cell Cycle, and Oxidative Stress in C6 Glioma Cell Line	Cell cultures: rat C6 GBM	The influence of proline extracellular concentration on GBM cultures was assessed. With increasing concentration: no cytotoxicity, no apoptosis, more ROS, and an increase in NF- κB—in general, a pro-proliferative influence.
Gönullu [37]	2012	Paraoxonase and Prolidase Activity in Patients with Malignant Gliomas	Serum of GBM patients	In patients diagnosed with GBM and anaplastic astrocytoma (grade 3), serum prolidase activity was lower than that in the healthy controls.
Verma et al. [38]	2018	Prolidase Activity and Oxidative Stress in Patients with Glioma	Serum and excised GBM tissue	In GBM patients, prolidase activity was elevated both in the serum and in tumor tissue.
Shao et al. [39]	2021	OIP5-AS1 specifies p53-driven POX transcription regulated by TRPC6 in glioma	Cell cultures, animal model	Molecular mechanism of how POX/PRODH is regulated by p53 through TRPC6. In general, POX/PRODH suppressed glioma. POX/PRODH in glioma was sixfold lower than that in the brain.
Zhao et al. [40]	2016	Metabolomics profiling in plasma samples from glioma patients correlates with tumor phenotypes	Database of plasma metabolomics	Metabolic pathways and metabolites of proline and arginine had the highest impact in differentiating high vs. low grade, IDH mutation vs. wild type.
Huang et al. [41]	2017	A prospective study of serum metabolites and glioma risk	Plasma of GBM patients and matched controls	Proline/arginine metabolism related to malignant glioma: lower metabolite concentration in the plasma of GBM patients.
Björkblom et al. [42]	2020	Metabolic response patterns in brain microdialysis fluids and serum during interstitial cisplatin treatment of high-grade glioma	Brain and tumor microdialysis fluid	Compared with the unaffected brain, glioma microdialysis fluid had a 4.4-fold higher proline concentration.
Jonsson et al. [43]	2020	Identification of Pre-Diagnostic Metabolic Patterns for Glioma Using Subset Analysis of Matched Repeated Time Points	Plasma sample metabolomic profile	A study in a large biobank of plasma samples; selected glioma cases prior to diagnosis were matched with controls. Along with a number of other amino acids, plasma proline concentration in glioma patients was significantly higher even prior to diagnosis, with potential to consider proline as a pre-diagnostic glioma biomarker.
Panosyan et al. [44]	2017	In search of druggable targets for GBM amino acid metabolism	Database of gene expression	In large datasets of gene expression, the authors found that POX/PRODH gene expression was lower in GBM than in non-GBM. Among GBM samples, there was a 50/50 equilibrium in high vs. low POX/PRODH expression, and overall survival (OS) was shorter in high-POX/PRODH patients, with a high level of significance. Although significant, the authors reflected upon POX/PRODH somewhat superficially. Additionally, the authors emphasized the role of glutamate.
Prabhu et al. [45]	2019	Integrative cross-platform analyses identify enhanced heterotrophy as a metabolic hallmark in glioblastoma	GBM and low-grade glioma tissues metabolomic and genomic profiles	In a search for a glioma metabolic reprogramming program, the authors found that proline metabolism was significantly altered.
Loreck et al. [46]	1987	Regulation of the pentose phosphate pathway in human astrocytes and gliomas	Cells cultures	A historical study. Proline cycle in gliomas was absent; no effect was observed on glucose metabolism due to the addition of proline to the medium: “POX is low to absent”.
Lefauconnier et al. [47]	1976	Free Amino Acids and Related Substances in Human Glial Tumours and in Fetal Brain–Comparison with Normal Adult Brain	Tissues: excised GBM, fetal and adult brain	A historical study. Proline concentration in GBM was 10x higher than that in the brain.

## References

[1] Ostrom Q.T., Gittleman H., Farah P., Ondracek A., Chen Y., Wolinsky Y., Stroup N.E., Kruchko C., Barnholtz-Sloan J.S. (2013). CBTRUS statistical report: Primary brain and central nervous system tumors diagnosed in the United States in 2006–2010. Neuro Oncol..

[2] Louis D.N., Perry A., Wesseling P., Brat D.J., Cree I.A., Figarella-Branger D., Hawkins C., Ng H.K., Pfister S.M., Reifenberger G. (2021). The 2021 WHO Classification of Tumors of the Central Nervous System: A summary. Neuro Oncol..

[3] Avgeropoulos N.G., Batchelor T.T. (1999). New treatment strategies for malignant gliomas. Oncologist.

[4] Tan A.C., Ashley D.M., López G.Y., Malinzak M., Friedman H.S., Khasraw M. (2020). Management of glioblastoma: State of the art and future directions. CA Cancer J. Clin..

[5] Stylli S.S. (2020). Novel Treatment Strategies for Glioblastoma. Cancers.

[6] Tanner J.J., Fendt S.M., Becker D.F. (2018). The Proline Cycle as a Potential Cancer Therapy Target. Biochemistry.

[7] Liu W., Hancock C.N., Fischer J.W., Harman M., Phang J.M. (2015). Proline biosynthesis augments tumor cell growth and aerobic glycolysis: Involvement of pyridine nucleotides. Sci. Rep..

[8] Burke L., Guterman I., Palacios Gallego R., Britton R.G., Burschowsky D., Tufarelli C., Rufini A. (2020). The Janus-like role of proline metabolism in cancer. Cell Death Discov..

[9] Grassian A.R., Parker S.J., Davidson S.M., Divakaruni A.S., Green C.R., Zhang X., Slocum K.L., Pu M., Lin F., Vickers C. (2014). IDH1 mutations alter citric acid cycle metabolism and increase dependence on oxidative mitochondrial metabolism. Cancer Res..

[10] Bhavya B., Anand C.R., Madhusoodanan U.K., Rajalakshmi P., Krishnakumar K., Easwer H.V., Deepti A.N., Gopala S. (2020). To be Wild or Mutant: Role of Isocitrate Dehydrogenase 1 (IDH1) and 2-Hydroxy Glutarate (2-HG) in Gliomagenesis and Treatment Outcome in Glioma. Cell. Mol. Neurobiol..

[11] Maus A., Peters G.J. (2017). Glutamate and α-ketoglutarate: Key players in glioma metabolism. Amino Acids.

[12] Tapiero H., Mathé G., Couvreur P., Tew K.D. (2002). II. Glutamine and glutamate. Biomed. Pharmacother..

[13] Phang J.M., Liu W. (2012). Proline metabolism and cancer. Front. Biosci..

[14] Phang J.M., Liu W., Hancock C., Christian K.J. (2012). The proline regulatory axis and cancer. Front. Oncol..

[15] Phang J.M. (2019). Proline Metabolism in Cell Regulation and Cancer Biology: Recent Advances and Hypotheses. Antioxid. Redox Signal..

[16] Misiura M., Miltyk W. (2020). Current Understanding of the Emerging Role of Prolidase in Cellular Metabolism. Int. J. Mol. Sci..

[17] Lupi A., Tenni R., Rossi A., Cetta G., Forlino A. (2008). Human prolidase and prolidase deficiency: An overview on the characterization of the enzyme involved in proline recycling and on the effects of its mutations. Amino Acids.

[18] Spodenkiewicz M., Cleary M., Massier M., Fitsialos G., Cottin V., Jouret G., Poirsier C., Doco-Fenzy M., Lèbre A.S. (2020). Clinical Genetics of Prolidase Deficiency: An Updated Review. Biology.

[19] Eni-Aganga I., Lanaghan Z.M., Balasubramaniam M., Dash C., Pandhare J. (2021). PROLIDASE: A Review from Discovery to its Role in Health and Disease. Front. Mol. Biosci..

[20] Phang J.M., Donald S.P., Pandhare J., Liu Y. (2008). The metabolism of proline, a stress substrate, modulates carcinogenic pathways. Amino Acids.

[21] Phang J.M., Liu W., Hancock C.N., Fischer J.W. (2015). Proline metabolism and cancer: Emerging links to glutamine and collagen. Curr. Opin. Clin. Nutr. Metab. Care.

[22] Phang J.M., Pandhare J., Liu Y. (2008). The metabolism of proline as microenvironmental stress substrate. J. Nutr..

[23] Liang X., Zhang L., Natarajan S.K., Becker D.F. (2013). Proline mechanisms of stress survival. Antioxid. Redox Signal..

[24] Bogner A.N., Stiers K.M., Tanner J.J. (2021). Structure, biochemistry, and gene expression patterns of the proline biosynthetic enzyme pyrroline-5-carboxylate reductase (PYCR), an emerging cancer therapy target. Amino Acids.

[25] Du J., Zhu S., Lim R.R., Chao J.R. (2021). Proline metabolism and transport in retinal health and disease. Amino Acids.

[26] Obara-Michlewska M., Szeliga M. (2020). Targeting Glutamine Addiction in Gliomas. Cancers.

[27] Li P., Wu G. (2018). Roles of dietary glycine, proline, and hydroxyproline in collagen synthesis and animal growth. Amino Acids.

[28] Patriarca E.J., Cermola F., D’Aniello C., Fico A., Guardiola O., De Cesare D., Minchiotti G. (2021). The Multifaceted Roles of Proline in Cell Behavior. Front. Cell Dev. Biol..

[29] Yu X.C., Zhang W., Oldham A., Buxton E., Patel S., Nghi N., Tran D., Lanthorn T.H., Bomont C., Shi Z.C. (2009). Discovery and characterization of potent small molecule inhibitors of the high affinity proline transporter. Neurosci. Lett..

[30] Wyse A.T., Netto C.A. (2011). Behavioral and neurochemical effects of proline. Metab. Brain Dis..

[31] Orešič M., Tang J., Seppänen-Laakso T., Mattila I., Saarni S.E., Saarni S.I., Lönnqvist J., Sysi-Aho M., Hyötyläinen T., Perälä J. (2011). Metabolome in schizophrenia and other psychotic disorders: A general population-based study. Genome Med..

[32] Schafer I.A., Scriver C.R., Efron M.L. (1962). Familial hyperprolinemia, cerebral dysfunction and renal anomalies occurring in a family with hereditary nephropathy and deafness. N. Engl. J. Med..

[33] Mitsubuchi H., Nakamura K., Matsumoto S., Endo F. (2008). Inborn errors of proline metabolism. J. Nutr..

[34] Hollinshead K.E.R., Munford H., Eales K.L., Bardella C., Li C., Escribano-Gonzalez C., Thakker A., Nonnenmacher Y., Kluckova K., Jeeves M. (2018). Oncogenic IDH1 Mutations Promote Enhanced Proline Synthesis through PYCR1 to Support the Maintenance of Mitochondrial Redox Homeostasis. Cell Rep..

[35] Cappelletti P., Tallarita E., Rabattoni V., Campomenosi P., Sacchi S., Pollegioni L. (2018). Proline oxidase controls proline, glutamate, and glutamine cellular concentrations in a U87 glioblastoma cell line. PLoS ONE.

[36] Ferreira A.G.K., Biasibetti-Brendler H., Sidegum D.S.V., Loureiro S.O., Figueiró F., Wyse A.T.S. (2021). Effect of Proline on Cell Death, Cell Cycle, and Oxidative Stress in C6 Glioma Cell Line. Neurotox. Res..

[37] Gonullu E., Silav G., Kaya M., Arslan M., Gonullu H., Arslan H., Cebi A., Demir H. (2012). Paraoxonase and Prolidase Activity in Patietns With Malignant Gliomas. J. Neurol. Sci.-Turk..

[38] Verma A., Singh K., Pandey S., Srivastava R. (2018). Prolidase Activity and Oxidative Stress in Patients with Glioma. J. Clin. Diagn. Res..

[39] Shao W., Hao Z.Y., Chen Y.F., Du J., He Q., Ren L.L., Gao Y., Song N., Song Y., He H. (2021). OIP5-AS1 specifies p53-driven POX transcription regulated by TRPC6 in glioma. J. Mol. Cell Biol..

[40] Zhao H., Heimberger A.B., Lu Z., Wu X., Hodges T.R., Song R., Shen J. (2016). Metabolomics profiling in plasma samples from glioma patients correlates with tumor phenotypes. Oncotarget.

[41] Huang J., Weinstein S.J., Kitahara C.M., Karoly E.D., Sampson J.N., Albanes D. (2017). A prospective study of serum metabolites and glioma risk. Oncotarget.

[42] Björkblom B., Jonsson P., Tabatabaei P., Bergström P., Johansson M., Asklund T., Bergenheim A.T., Antti H. (2020). Metabolic response patterns in brain microdialysis fluids and serum during interstitial cisplatin treatment of high-grade glioma. Br. J. Cancer.

[43] Jonsson P., Antti H., Späth F., Melin B., Björkblom B. (2020). Identification of Pre-Diagnostic Metabolic Patterns for Glioma Using Subset Analysis of Matched Repeated Time Points. Cancers.

[44] Panosyan E.H., Lin H.J., Koster J., Lasky J.L. (2017). In search of druggable targets for GBM amino acid metabolism. BMC Cancer.

[45] Prabhu A.H., Kant S., Kesarwani P., Ahmed K., Forsyth P., Nakano I., Chinnaiyan P. (2019). Integrative cross-platform analyses identify enhanced heterotrophy as a metabolic hallmark in glioblastoma. Neuro Oncol..

[46] Loreck D.J., Galarraga J., Van der Feen J., Phang J.M., Smith B.H., Cummins C.J. (1987). Regulation of the pentose phosphate pathway in human astrocytes and gliomas. Metab. Brain Dis..

[47] Lefauconnier J.M., Portemer C., Chatagner F. (1976). Free amino acids and related substances in human glial tumours and in fetal brain: Comparison with normal adult brain. Brain Res..

[48] Hagedorn C.H., Phang J.M. (1983). Transfer of reducing equivalents into mitochondria by the interconversions of proline and delta 1-pyrroline-5-carboxylate. Arch. Biochem. Biophys..

[49] Hagedorn C.H., Phang J.M. (1986). Catalytic transfer of hydride ions from NADPH to oxygen by the interconversions of proline and delta 1-pyrroline-5-carboxylate. Arch. Biochem. Biophys..

[50] Surazynski A., Miltyk W., Palka J., Phang J.M. (2008). Prolidase-dependent regulation of collagen biosynthesis. Amino Acids.

[51] Insolia V., Priori E.C., Gasperini C., Coppa F., Cocchia M., Iervasi E., Ferrari B., Besio R., Maruelli S., Bernocchi G. (2020). Prolidase enzyme is required for extracellular matrix integrity and impacts on postnatal cerebellar cortex development. J. Comp. Neurol..

[52] Insolia V., Piccolini V.M. (2014). Brain morphological defects in prolidase deficient mice: First report. Eur. J. Histochem..

[53] Payne L.S., Huang P.H. (2013). The pathobiology of collagens in glioma. Mol. Cancer Res..

[54] Mughal A.A., Zhang L., Fayzullin A., Server A., Li Y., Wu Y., Glass R., Meling T., Langmoen I.A., Leergaard T.B. (2018). Patterns of Invasive Growth in Malignant Gliomas-The Hippocampus Emerges as an Invasion-Spared Brain Region. Neoplasia.

[55] Inda M.M., Bonavia R., Seoane J. (2014). Glioblastoma multiforme: A look inside its heterogeneous nature. Cancers.

[56] Di Nunno V., Franceschi E., Tosoni A., Gatto L., Lodi R., Bartolini S., Brandes A.A. (2021). Glioblastoma: Emerging Treatments and Novel Trial Designs. Cancers.

[57] Saadeh F.S., Mahfouz R., Assi H.I. (2018). EGFR as a clinical marker in glioblastomas and other gliomas. Int. J. Biol. Markers.

[58] Gao Y., Vallentgoed W.R., French P.J. (2018). Finding the Right Way to Target EGFR in Glioblastomas; Lessons from Lung Adenocarcinomas. Cancers.

[59] Pearson J.R.D., Regad T. (2017). Targeting cellular pathways in glioblastoma multiforme. Signal Transduct. Target. Ther..

[60] Yang L., Li Y., Bhattacharya A., Zhang Y. (2017). PEPD is a pivotal regulator of p53 tumor suppressor. Nat. Commun..

[61] Surazynski A., Donald S.P., Cooper S.K., Whiteside M.A., Salnikow K., Liu Y., Phang J.M. (2008). Extracellular matrix and HIF-1 signaling: The role of prolidase. Int. J. Cancer.

[62] Zareba I., Palka J. (2016). Prolidase-proline dehydrogenase/proline oxidase-collagen biosynthesis axis as a potential interface of apoptosis/autophagy. Biofactors.

[63] Wang G., Wang J.J., Fu X.L., Guang R., To S.T. (2017). Advances in the targeting of HIF-1α and future therapeutic strategies for glioblastoma multiforme (Review). Oncol. Rep..

[64] Kaur B., Khwaja F.W., Severson E.A., Matheny S.L., Brat D.J., Van Meir E.G. (2005). Hypoxia and the hypoxia-inducible-factor pathway in glioma growth and angiogenesis. Neuro Oncol..

[65] Garcia-Fabiani M.B., Haase S., Comba A., Carney S., McClellan B., Banerjee K., Alghamri M.S., Syed F., Kadiyala P., Nunez F.J. (2021). Genetic Alterations in Gliomas Remodel the Tumor Immune Microenvironment and Impact Immune-Mediated Therapies. Front. Oncol..

[66] Li W., Holsinger R.M., Kruse C.A., Flügel A., Graeber M.B. (2013). The potential for genetically altered microglia to influence glioma treatment. CNS Neurol. Disord. Drug Targets.

[67] Kim J.K., Jin X., Sohn Y.W., Jeon H.Y., Kim E.J., Ham S.W., Jeon H.M., Chang S.Y., Oh S.Y., Yin J. (2014). Tumoral RANKL activates astrocytes that promote glioma cell invasion through cytokine signaling. Cancer Lett..

[68] Han J., Alvarez-Breckenridge C.A., Wang Q.E., Yu J. (2015). TGF-β signaling and its targeting for glioma treatment. Am. J. Cancer Res..

[69] D’Aniello C., Patriarca E.J., Phang J.M., Minchiotti G. (2020). Proline Metabolism in Tumor Growth and Metastatic Progression. Front. Oncol..

[70] Liu W., Glunde K., Bhujwalla Z.M., Raman V., Sharma A., Phang J.M. (2012). Proline oxidase promotes tumor cell survival in hypoxic tumor microenvironments. Cancer Res..

[71] Liu Y., Mao C., Liu S., Xiao D., Shi Y., Tao Y. (2021). Proline dehydrogenase in cancer: Apoptosis, autophagy, nutrient dependency and cancer therapy. Amino Acids.

[72] Zareba I., Celinska-Janowicz K., Surazynski A., Miltyk W., Palka J. (2018). Proline oxidase silencing induces proline-dependent pro-survival pathways in MCF-7 cells. Oncotarget.

[73] Rinaldi M., Caffo M., Minutoli L., Marini H., Abbritti R.V., Squadrito F., Trichilo V., Valenti A., Barresi V., Altavilla D. (2016). ROS and Brain Gliomas: An Overview of Potential and Innovative Therapeutic Strategies. Int. J. Mol. Sci..

[74] Liu W., Phang J.M. (2012). Proline dehydrogenase (oxidase) in cancer. Biofactors.

[75] Annibali D., Whitfield J.R., Favuzzi E., Jauset T., Serrano E., Cuartas I., Redondo-Campos S., Folch G., Gonzàlez-Juncà A., Sodir N.M. (2014). Myc inhibition is effective against glioma and reveals a role for Myc in proficient mitosis. Nat. Commun..

[76] Tateishi K., Iafrate A.J., Ho Q., Curry W.T., Batchelor T.T., Flaherty K.T., Onozato M.L., Lelic N., Sundaram S., Cahill D.P. (2016). Myc-Driven Glycolysis Is a Therapeutic Target in Glioblastoma. Clin. Cancer Res..

[77] Liu Y., Borchert G.L., Donald S.P., Diwan B.A., Anver M., Phang J.M. (2009). Proline oxidase functions as a mitochondrial tumor suppressor in human cancers. Cancer Res..

[78] Wang C.Y., Chiao C.C., Phan N.N., Li C.Y., Sun Z.D., Jiang J.Z., Hung J.H., Chen Y.L., Yen M.C., Weng T.Y. (2020). Gene signatures and potential therapeutic targets of amino acid metabolism in estrogen receptor-positive breast cancer. Am. J. Cancer Res..

[79] Zareba I., Surazynski A., Chrusciel M., Miltyk W., Doroszko M., Rahman N., Palka J. (2017). Functional Consequences of Intracellular Proline Levels Manipulation Affecting PRODH/POX-Dependent Pro-Apoptotic Pathways in a Novel in Vitro Cell Culture Model. Cell Physiol. Biochem..

[80] Liu Y., Borchert G.L., Surazynski A., Phang J.M. (2008). Proline oxidase, a p53-induced gene, targets COX-2/PGE2 signaling to induce apoptosis and inhibit tumor growth in colorectal cancers. Oncogene.

[81] Tołoczko-Iwaniuk N., Dziemiańczyk-Pakieła D., Celińska-Janowicz K., Zaręba I., Klupczyńska A., Kokot Z.J., Nowaszewska B.K., Reszeć J., Borys J., Miltyk W. (2020). Proline-Dependent Induction of Apoptosis in Oral Squamous Cell Carcinoma (OSCC)-The Effect of Celecoxib. Cancers.

[82] Celińska-Janowicz K., Zaręba I., Lazarek U., Teul J., Tomczyk M., Pałka J., Miltyk W. (2018). Constituents of propolis: Chrysin, caffeic acid, p-coumaric acid, and ferulic acid induce PRODH/POX-dependent apoptosis in human tongue squamous cell carcinoma cell (CAL-27). Front. Pharmacol..

[83] Olivares O., Mayers J.R., Gouirand V., Torrence M.E., Gicquel T., Borge L., Lac S., Roques J., Lavaut M.N., Berthezène P. (2017). Collagen-derived proline promotes pancreatic ductal adenocarcinoma cell survival under nutrient limited conditions. Nat. Commun..

[84] Liu Y., Mao C., Wang M., Liu N., Ouyang L., Liu S., Tang H., Cao Y., Wang X., Xiao D. (2020). Cancer progression is mediated by proline catabolism in non-small cell lung cancer. Oncogene.

[85] Yan Y., Chang L., Tian H., Wang L., Zhang Y., Yang T., Li G., Hu W., Shah K., Chen G. (2018). 1-Pyrroline-5-carboxylate released by prostate Cancer cell inhibit T cell proliferation and function by targeting SHP1/cytochrome c oxidoreductase/ROS Axis. J. Immunother. Cancer.

[86] Elia I., Broekaert D., Christen S., Boon R., Radaelli E., Orth M.F., Verfaillie C., Grünewald T.G.P., Fendt S.M. (2017). Proline metabolism supports metastasis formation and could be inhibited to selectively target metastasizing cancer cells. Nat. Commun..

[87] Fang H., Du G., Wu Q., Liu R., Chen C., Feng J. (2019). HDAC inhibitors induce proline dehydrogenase (POX) transcription and anti-apoptotic autophagy in triple negative breast cancer. Acta Biochim. Biophys. Sin..

[88] Maxwell S.A., Rivera A. (2003). Proline oxidase induces apoptosis in tumor cells, and its expression is frequently absent or reduced in renal carcinomas. J. Biol. Chem..

[89] Nilsson R., Jain M., Madhusudhan N., Sheppard N.G., Strittmatter L., Kampf C., Huang J., Asplund A., Mootha V.K. (2014). Metabolic enzyme expression highlights a key role for MTHFD2 and the mitochondrial folate pathway in cancer. Nat. Commun..

[90] Wang D., Wang L., Zhang Y., Yan Z., Liu L., Chen G. (2019). PYCR1 promotes the progression of non-small-cell lung cancer under the negative regulation of miR-488. Biomed. Pharmacother..

[91] Cai F., Miao Y., Liu C., Wu T., Shen S., Su X., Shi Y. (2018). Pyrroline-5-carboxylate reductase 1 promotes proliferation and inhibits apoptosis in non-small cell lung cancer. Oncol. Lett..

[92] Sang S., Zhang C., Shan J. (2019). Pyrroline-5-Carboxylate Reductase 1 Accelerates the Migration and Invasion of Nonsmall Cell Lung Cancer. Cancer Biother. Radiopharm..

[93] Gao Y., Luo L., Xie Y., Zhao Y., Yao J., Liu X. (2020). PYCR1 knockdown inhibits the proliferation, migration, and invasion by affecting JAK/STAT signaling pathway in lung adenocarcinoma. Mol. Carcinog..

[94] Ding J., Kuo M.L., Su L., Xue L., Luh F., Zhang H., Wang J., Lin T.G., Zhang K., Chu P. (2017). Human mitochondrial pyrroline-5-carboxylate reductase 1 promotes invasiveness and impacts survival in breast cancers. Carcinogenesis.

[95] Zhuang J., Song Y., Ye Y., He S., Ma X., Zhang M., Ni J., Wang J., Xia W. (2019). PYCR1 interference inhibits cell growth and survival via c-Jun N-terminal kinase/insulin receptor substrate 1 (JNK/IRS1) pathway in hepatocellular cancer. J. Transl. Med..

[96] Ding Z., Ericksen R.E., Escande-Beillard N., Lee Q.Y., Loh A., Denil S., Steckel M., Haegebarth A., Wai Ho T.S., Chow P. (2020). Metabolic pathway analyses identify proline biosynthesis pathway as a promoter of liver tumorigenesis. J. Hepatol..

[97] Zeng T., Zhu L., Liao M., Zhuo W., Yang S., Wu W., Wang D. (2017). Knockdown of PYCR1 inhibits cell proliferation and colony formation via cell cycle arrest and apoptosis in prostate cancer. Med. Oncol..

[98] Xiao S., Li S., Yuan Z., Zhou L. (2020). Pyrroline-5-carboxylate reductase 1 (PYCR1) upregulation contributes to gastric cancer progression and indicates poor survival outcome. Ann. Transl. Med..

[99] Du S., Sui Y., Ren W., Zhou J., Du C. (2021). PYCR1 promotes bladder cancer by affecting the Akt/Wnt/β-catenin signaling. J. Bioenerg. Biomembr..

[100] Ye Y., Wu Y., Wang J. (2018). Pyrroline-5-carboxylate reductase 1 promotes cell proliferation via inhibiting apoptosis in human malignant melanoma. Cancer Manag. Res..

[101] Wilk P., Wątor E., Weiss M.S. (2021). Prolidase—A protein with many faces. Biochimie.

[102] Celik S., Kızıltan R., Yılmaz E.M., Yılmaz Ö., Demir H. (2017). Potential diagnostic and prognostic significance of plasma prolidase activity in gastric cancer. Biomark. Med..

[103] Cechowska-Pasko M., Pałka J., Wojtukiewicz M.Z. (2006). Enhanced prolidase activity and decreased collagen content in breast cancer tissue. Int. J. Exp. Pathol..

[104] Arioz D.T., Camuzcuoglu H., Toy H., Kurt S., Celik H., Aksoy N. (2009). Serum prolidase activity and oxidative status in patients with stage I endometrial cancer. Int. J. Gynecol. Cancer.

[105] Gecit I., Aslan M., Gunes M., Pirincci N., Esen R., Demir H., Ceylan K. (2012). Serum prolidase activity, oxidative stress, and nitric oxide levels in patients with bladder cancer. J. Cancer Res. Clin. Oncol..

[106] Guszczyn T., Sobolewski K. (2004). Deregulation of collagen metabolism in human stomach cancer. Pathobiology.

[107] Sayır F., Şehitoğulları A., Demir H., Aslan M., Çobanoğlu U., Bilgin C. (2019). Serum prolidase activity, total oxidant/antioxidant, and nitric oxide levels in patients with esophageal squamous cell carcinoma. Turk. J. Thorac. Cardiovasc. Surg..

[108] Palka J., Surazynski A., Karna E., Orlowski K., Puchalski Z., Pruszynski K., Laszkiewicz J., Dzienis H. (2002). Prolidase activity disregulation in chronic pancreatitis and pancreatic cancer. Hepatogastroenterology.

